# Nucleomodulins from gut bacteria: diverse mechanisms of translocation and interaction with host nuclear processes

**DOI:** 10.1128/aem.00211-25

**Published:** 2025-07-18

**Authors:** Sania Korgaonkar, Chandrani Bose, Swadha Anand

**Affiliations:** 1 TCS Research, Tata Consultancy Services Limited 483340 , Pune, Maharashtra, India; University of Illinois Urbana-Champaign, Urbana, Illinois, USA

**Keywords:** nucleomodulins, gut pathogens, gut microbiome, nuclear localization signals, virulence factors, gene expression

## Abstract

Nucleomodulins (NMs) are bacterial nuclear-targeted effector proteins that interfere with a multitude of host cellular processes to shield pathogens from host immune responses. Recent years observed a surge in NM-related research owing to their potential role in both infectious diseases and cancer development. However, considering the complex nature of the interaction between NM and host factors, the field of “NM-disease axis” is still in the nascent phase. Thus, a comprehensive view of the known mechanisms of translocation of NMs to the host cell nucleus and mode of action, thereafter, is crucial toward deeper exploration of the “NM-disease axis.” The human gut is the major host niche to harbor bacterial cells (as part of “gut microbiota”). The current review provides an extensive collation of nucleomodulin-mediated mechanisms employed by opportunistic gut pathogens. The insights from the review would help in designing future experiments toward utilizing the NM-associated host-pathogen interaction modules in disease diagnostics and therapy.

## INTRODUCTION

Bacterial pathogens possess a dynamic ability in adapting to highly sophisticated mechanisms to counter the complex network of immune responses produced by the host (1, 2). The success of a pathogen is governed by continual variation in the strategies employed by the bacterium in an attempt to persist intracellularly and spread infection. Some examples of such strategies include bacterial cell surface modifications, release of toxins, alterations in antigenic properties, and mimicking of host proteins (2). This review is based on one such pathogenic adaptation that involves secretion of bacterial effector molecules called nucleomodulins (NMs). The uniqueness of nucleomodulins lies in their potential to access the host cell nucleus, appositely known as the control center of the cell, unlike the widely discussed pathogenic strategies modulating host cytosolic pathways (3, 4).

The term “nucleomodulins” was coined in 2012 by Bierne and Cossart (5). As the name implies, NMs refers to bacterial molecules that have the potential to translocate to the host nucleus (“nucleo”) and modulate (“modulins”) host cell responses, which may have a long-term genetic or epigenetic effect on the host (5, 6). Most early reports on such bacterial molecules pertain to the phytopathogen, *Agrobacterium tumefaciens*, which is a causal agent for crown gall tumor in plants and is known to transfer and integrate bacterial plasmid DNA into the host genome (7). In the context of the human host, certain opportunistic pathogens have been reported to harbor nucleomodulins that interfere with host cellular functions, such as cell cycle processes, cell signaling pathways, and chromatin remodeling, leading to alterations in the transcriptional networks, as well as the physical state of the nuclei (3, 5, 6).

One of the commonly discussed mechanisms of nucleomodulin translocation involves mimicking the strategies employed by human nuclear-targeted proteins. This primarily includes harboring eukaryotic nuclear localization signals (NLS), which are short peptide sequences that facilitate the transport of proteins to the nucleus through the nuclear pore complex (NPC), enabling them to traverse across the nuclear envelope (8). Depending on the amino acid sequence of this motif, NLSs are classified broadly into two types, classical (cNLS) and non-classical NLS (ncNLS) (9–11). cNLS are categorized into monopartite (MP) signals, which consist of 4–8 basic amino acids like arginine (R) and lysine (K), and “bipartite” (BP) signals, which comprise two clusters of basic amino acids separated by a linker amino acid sequence. The import of NMs into the host nucleus is facilitated by “importins,” which belong to a class of proteins known as “karyopherins” (9, 10). In classical import, NLS is recognized by importin α, which binds to the cargo and presents it to importin β that carries the complex into the host nucleus. Classical signals are further classified into six classes based on their binding properties with importin α (12). NLS that do not resemble the canonical classical signals are termed as ncNLS (13, 14). One example is “PY-NLS,” which features a distinct R/K/H(X)_2-5_PY (where X_2-5_ represents any sequence consisting of 2 to 5 amino acid residues) motif located at the C-terminus. Another example includes NLS of Rex protein of human T-cell leukemia virus type 1, which comprises long stretches of arginine residues (14, 15). In both of these cases, NLS is directly recognized by importin β, bypassing the initial recognition by the adapter protein importin α, which is a characteristic of cNLS transport (14, 15). In addition to the aforementioned mimicking strategies employed by NMs, the ones lacking NLS have been reported to either diffuse through the nuclear pores (MW <50 kDa) or hijack eukaryotic proteins trafficked to the host nucleus. However, these mechanisms are just a few of the several untapped strategies employed by the pathogen that require further exploration (8, 15).

Understanding these bacterial virulence mechanisms is crucial in the context of “host-microbiome interaction.” “Microbiome” refers to the entire genetic make-up of the microbial community (or “microbiota”) that reside in a particular niche (16). Our “gut microbiota” comprises of ~1,000 resident bacterial species (along with other microbial species) with around three million genes, out of which a fraction is capable of transitioning into opportunistic pathogens (17–20). These pathogens may utilize nucleomodulin-mediated mechanisms to interfere with host pathways. In view of this, the current review aims to collate information on nucleomodulins from gut bacteria and various mechanisms employed by these proteins to manipulate a multitude of host cell signals in favor of pathogen survival. Such a comprehensive view will deepen our understanding about the capabilities of gut pathogens in controlling the host nucleus and orchestrating mechanisms that lead to cell damage. This, in turn, could potentially facilitate the design of disease management strategies to effectively combat infections by manipulating the proteins that hamper the functioning of the cell’s command center.

## GUT BACTERIA AND NUCLEOMODULINS

**Fig 1:**
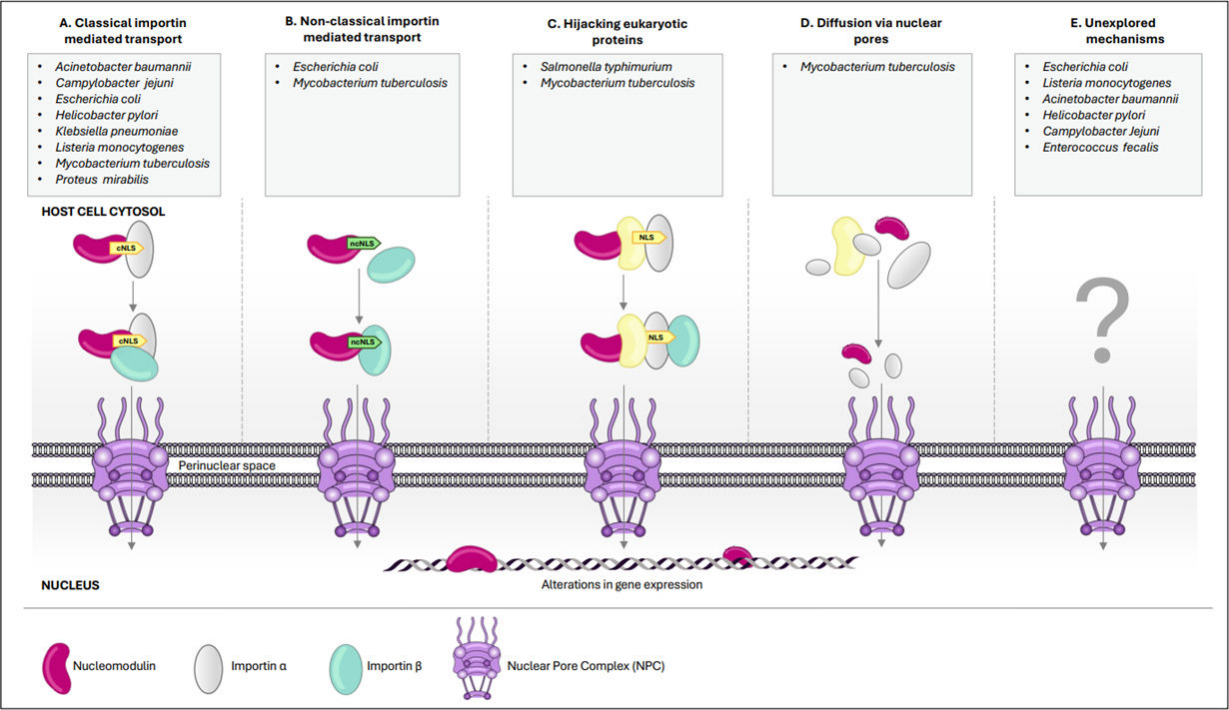
Different strategies employed by bacterial nucleomodulins to enter the host nucleus. (A) In classical importin-mediated transport, nucleomodulins (NM) comprising classical nuclear localization signal (cNLS) are recognized by importin α. This complex is then carried via nuclear pore complex (NPC) by importin β. (B) In non-classical importin-mediated transport, NMs comprising a non-classical NLS are recognized directly by importin β, thus mediating the transport across NPC. (C) Bacterial proteins hijack eukaryotic nuclear-targeted proteins having NLS, thereby gaining entry to the nucleus. (D) NMs with molecular weight <50 kDa enter the host nucleus by passive diffusion through NPC. (E) Unexplored mechanisms.

Recent literature has demonstrated the presence of nucleomodulins in multiple gut pathogens including *Salmonella typhimurium*, *Klebsiella pneumoniae*, *Helicobacter pylori*, and *Escherichia coli* (20–23). The identification of nucleomodulins *in vitro* typically involves fluorescent tagging, enabling the visualization of proteins translocated to the nucleus for initial screening. Additional experimental approaches are often necessary to elucidate the specific mechanisms used by nucleomodulins to enter the host nucleus and to determine the functional consequences on cellular processes (24). Various computational tools are also available for *in silico* identification of cNLSs such as NLStradamus, NLS Mapper, NucImport, and NucPred (25–28). Furthermore, studies involving site-directed mutagenesis have been performed to explore the significance of NLS in the interaction of nucleomodulins with other proteins (24). Table 1 provides a summary of NLSs found in various gut pathogens. The characteristics of NLSs, the mechanism of translocation of NMs (secreted by different gut bacteria) to the host cell nucleus (schematically shown in Fig. 1), and the mode of action thereafter have been discussed in detail in the following sections.

**TABLE 1:** Summary of nucleomodulins secreted by opportunistic gut pathogens^*a*^

Pathogen	Nucleomodulin (protein name, ID, or category)^*b*^	Mode of entering the nucleus	Method used for probing nuclear entry	^Start^NLS sequence^End^	Signal type	Mode of action (reference)
*Acinetobacter baumannii*	OmpA	Using NLS	Site-directed mutagenesis	^320^KTKEGRAMNRR^330^	MP and cNLS	Hinders physiological state of the cell, causing cell death (29)
	Tnp	Using NLS	Site-directed mutagenesis	^225^RKRKRK^230^	MP and cNLS	Suppresses E-cadherin expression by inducing DNA methylation of CpG islands in the promoter region (30)
*Campylobacter jejuni*	Cas9	Using NLS	Signal deletion	^44^RRLARSARKRLARRKAR^60^	BP and cNLS	Induces DNA damage (31, 32)
	CdtB	Unknown	–	–^*c*^	–	Cell-cycle arrest at G2/M ^50^
*Enterococcus faecalis*	Whole bacterial cell	Unknown	Fluorescence-activated cell sorting (FACS)	–	–	Suppresses NF-κB activation in macrophages (33)
*Escherichia coli*	CdtB	Using NLS	Site-directed mutagenesis	^195^REPADLEMNLTVPVRR^210^	BP and cNLS	Induced DNA damage, interrupts the cell cycle (23)
				^253^RRTQISSDHFPVGVSRR^269^		
	Cif	Unknown	–	–	–	Induces accumulation of the cyclin-dependent kinase inhibitors p21 and p27, causing cell cycle arrest (34, 35)
	EspF	51aa domain at the N-terminus	Site-directed mutagenesis	^21^SRVSSAGGTGFSVAPQAVRLTPVRVHSPFSPGSSNVNARTIFNVSSQVTS FTPS ^74^	ncNLS	EspF inflicts damage on the surrounding nucleolin, thereby affecting ribosome biogenesis (36)
	Tus	Using NLS	Site-directed mutagenesis	^227^KLKIKRPVK^235^	NLS-like sequence	Unknown
*Helicobacter pylori* 26695	HP0425	Using NLS	Site-directed mutagenesis	^3^KKELLKMSKKR^13^	MP and cNLS	Genomic DNA degradation by DNase I-like enzymatic activity (24)
	HP0059	Using NLS	Site-directed mutagenesis	^188^DKLKKL^193^	MP and cNLS	Genomic DNA degradation by DNase I-like enzymatic activity (22)
	Omp18	Using NLS	Site-directed mutagenesis	^57^PKKPKRKL^63^	MP and cNLS	Aids in virulence optimization for steady persistence of Hp in the host cell (37)
	Response regulator	Using NLS	Site-directed mutagenesis	^120^KKHPLEKPLKK^130^	BP and cNLS	Unknown (38)
	Secreted protein involved in flagellar motility	Using NLS	Site-directed mutagenesis	^66^KRKRWYELFKKKPK^79^	BP and cNLS	Unknown (38)
	UreA	Using NLS	Site-directed mutagenesis	^21^KKRKEK^26^	MP, cNLS	Regulates cell cycle progression by induction of hypoxia-induced factor −1α through TLR2 activation, leading to low levels of cyclin D1 (half-life) (39, 40)
*Klebsiella pneumoniae*	HsdM	Using NLS	Site-directed mutagenesis	^7^KKAKAKK^13^	MP, cNLS	Methylates eukaryotic DNA, unknown mechanisms (41)
*Listeria monocytogenes*	LntA	Using NLS	*In silico* identification	^122^IDAIKRSSEASADTEAFKKIFKEW^144^	BP and cNLS	Interferes with chromatin assembly by preventing recruitment of BAHD1 to ISGs, thereby inducing their expression (42)
	OrfX	Unknown	–	**–**	–	Targets RybP and reduces the oxidative capability of macrophages (43)
*Mycobacterium tuberculosis*	Rv1988	NLS-like sequence	Site-directed mutagenesis	^137^RR^139^, ^152^RR^154^, and ^169^RRRK^173^	–	Dimethylates arginine in histone H3, leading to suppression of genes implicated in inhibiting the action of macrophages (44)
	Rv0256c	Using cNLS	Site-directed mutagenesis	^473^RRRRPKIKQ^481^	MP and cNLS	Inhibits nitric oxide production in infected macrophages to thrive intracellularly (45, 46)
	Rv2966c	NLS-like sequence, via NPM1 shuttling protein (speculated)	Site-directed mutagenesis	–	–	Methylates non-CpG distinctly CpA and CpT dinucleotides of specific gene regions (47)
	Rv2067c	30 aa domain at the C-terminus	Signal deletion	–	–	Trimethylates H3K79 in free H3 histones, resulting in inhibition of caspase-dependent apoptosis (48)
	Rv3423.1	Diffusion through nuclear pores (speculated)	–	–	–	Regulation of genes by acetylating histone H3 at the K9/K14 positions (49)
*Porphyromonas gingivalis*	HRgpA	Unknown	–	–	–	Interferes with proteins implicated in cell cycle control processes (50)
*Proteus mirabilis*	Urease	Using cNLS	*In silico* identification	^22^RRLAKGLKLNYPEAVALISCAIMEGAREGKTVA^54^	BP, cNLS	Exerts pro-inflammatory effects on cells *in vitro* (51)
				^373^RTWQCAHKMKLQRGTLAGDSADNDNNRIKRYIAK^406^		
*Salmonella enterica*	SSph1	PKN1-assisted entry, lacks cNLS	–	–	–	Interacts with PKN1 to downregulate pro-inflammatory cytokine levels by inhibition of NF-κB-dependent gene expression (52)
	PipA GogA GtgA	Unknown	–	–	–	Cleave nuclear transcription factors RelA and RelB to modulate the NF-κB signaling pathway and influence host transcription (53)

^*a*^ BAHD1, bromo adjacent homology domain-containing 1; BP, bipartite; CdtB, cytolethal distending toxins; Cif, cycle-inhibiting factor; HRgpA, arginine-specific gingipain; ISGs, interferon-stimulated genes; LntA, Listeria nuclear targeted protein A; MP, monopartite; NF-κB, nuclear factor kappa-light-chain-enhancer of activated B cells; NLS, nuclear localization signal; NPM1, nucleophosmin; Omp, outer membrane protein; OrfX, open reading frame X; PKN1, protein kinase N1; RybP, RING1- and YY1-binding protein; SSph1, *Salmonella* secreted protein H1; TLR2, Toll-like receptor 2; Tnp, transposase; Tus, terminus utilization substance; TSGs, tumor suppressor genes.

^*b*^ In the case of *Enterococcus faecalis*, the whole bacterial cell acts as a nucleomodulin.

^*c*^ – denotes not applicable.

## SUPPRESSION OF CYTOKINE PRODUCTION BY *SALMONELLA TYPHIMURIUM*

*Salmonella typhimurium* is a non-typhoidal strain often associated with gastroenteritis (53). Proteins from this bacterium reported to be translocated to the nucleus include SSpH1, PipA, GtgA, and GogA (20, 53). SSpH1 is a Type III secretion system (T3SS)-mediated bacterial effector E3 ubiquitin ligase with LPX repeats (20, 54). In early reports, since SSpH1 lacks cNLS, it was hypothesized to enter the host nucleus with the help of eukaryotic protein kinase, PKN1. However, recent reports suggest the interaction of the SSph1 LRR domain with the HR1b subdomain of PKN1 in the cytoplasm, resulting in the ubiquitylation of PKN1 *in vitro*, thereby suppressing androgen receptor signaling (52). On another account, SspH1 enters the host cell nucleus and is known to suppress nuclear factor-kappa B (NF-κB)-dependent gene expression, resulting in downregulation of proinflammatory cytokine production (55). Another set of NMs from *S. typhimurium* includes GogA, GtgA, and PipA, which are homologous zinc metalloproteases that are part of the PipA family of effector proteins. These NMs proteolytically cleave transcription factors RelA and RelB, thereby deregulating NF-κB expression, resulting in attenuation of initial inflammatory responses. As a result, pathogens can persist in the system for extended durations. Sun et al. (53) demonstrated severe inflammation and increased virulence in a mouse model lacking GogA, GtgA, and PipA, thereby highlighting their role in maintaining virulence levels that do not induce inflammation (53, 56).

## HOST CELL DNA METHYLATION BY *KLEBSIELLA PNEUMONIAE*

*Klebsiella pneumoniae* is a well-established opportunistic, multidrug-resistant gut pathogen, commonly implicated in hospital-acquired urinary tract infections (21). *K. pneumoniae* encodes HsdM, a DNA methylase and a subunit of the Type I restriction modification system, which has been experimentally shown to be translocated to the host nucleus. This translocation is facilitated by an MP cNLS sequence ^7^KKAKAKK^13^, which is recognized by the eukaryotic importin α protein. Although HsdM was not found to critically damage eukaryotic DNA *in vitro* (41), detailed investigation of the biological relevance of DNA methylation by HsdM would be crucial for better understanding the virulence mechanisms of *K. pneumoniae*.

## NUCLEOMODULINS AIDING IN THE STEADY PERSISTENCE OF *HELICOBACTER PYLORI*

*Helicobacter pylori* has been implicated in around 90% of gastric cancer cases (57, 58). Cytotoxin-associated gene A (CagA) is a well-studied primary virulence factor associated with *H. pylori* infections. CagA, an oncoprotein involved in gastric adenocarcinomas in mammals (59), is directed to gastric epithelial cells with the help of the Type IV secretion system (T4SS). Once secreted by the bacterial cell, CagA anchors to the inner leaflet of the host cell’s plasma membrane, where it undergoes tyrosine phosphorylation, leading to a series of reactions that ultimately activate specific Wnt target genes (39, 59). While the main mechanism occurs in the cytoplasm, there have been reports of CagA being detected in the nucleus as well. However, its exact role in the nucleus remains poorly understood (60). Besides these mechanisms, two more proteins, HP0425 and HP0059, have been shown to harbor the MP cNLS sequence. These proteins with DNase I-like activity have been experimentally determined to enter the host nucleus and degrade host genomic DNA, resulting in subsequent cell death (22, 24). Further, *H. pylori* UreA, the structural subunit of urease, also contains a functional MP cNLS, which enables its entry into the host nucleus (38). Apart from *H. pylori* UreA, ureases in general have been suggested as virulent factors in various pathogenic bacteria, including *Staphylococcus* spp., *Ureaplasma urealyticum*, *Corynebacterium* spp., and *Klebsiella* spp. (40). *H. pylori* urease is an immunogen that is upregulated in patients with gastric cancer (61). During infection, it is delivered to the host cells via outer membrane vesicles, leading to the induction of hypoxia-inducible factor 1-alpha (HIF-1α) through TLR2 activation, thereby lowering levels of cyclin D1 and affecting cell cycle progression (62). Urease, typically a cytosolic enzyme, may also exert an influence within the host nucleus, potentially causing changes in the cell cycle beyond its primary physiological function of acid neutralization. Mutations in the UreA NLS were shown to impair the ability of the protein to enter the nucleus of cultured gastric epithelial cells, thereby causing UreA to be exclusively located in the cytoplasm (38). Lee et al. (63) demonstrated that UreA regulates a multitude of morphogenesis-related genes when present in the nucleus (63). Another bacterial urease reported to enter the host nucleus is observed in *Proteus mirabilis*, a microbe associated with urinary tract infections and also a commensal in the human gastrointestinal tract (64). Although *P. mirabilis* urease comprises two putative BP NLSs, the functionality of these NLSs has not yet been established. However, nuclear *P. mirabilis* urease has been reported to exert pro-inflammatory effects on the host cells *in vitro* (51).

Other *H. pylori* proteins translocated to the nucleus include outer membrane protein 18 (Omp18), transcriptional activator FlgR, and META domain-containing protein (38). Among these proteins, OMPs usually play a role in imparting virulence or assisting other virulent factors by facilitating adhesion for effective colonization inside the host (37, 65). Omp18, an antigenic protein expressed by most *H. pylori* strains, contains a monopartite cNLS that allows it to enter the host nucleus (38). In the nucleus, it was shown to modulate the levels of primary virulent factors, CagA and NapA, leading to a reduction in IFN-γ-mediated immune responses aimed at clearing the pathogen (37). In addition to Omp18, OMPs in *H. pylori*, such as SabA, OipA, and HopQ, are also recognized for their role in promoting bacterial pathogenesis. The expression of these virulence factors is regulated in response to the gastric environment (65). In-depth analysis of the aforementioned proteins may reveal additional regulatory mechanisms or novel pathways employed by *H. pylori* that contribute to promoting gastric metastasis.

## CELL CYCLE CONTROL BY *ESCHERICHIA COLI*, *CAMPYLOBACTER JEJUNI,* AND *PORPHYROMONAS GINGIVALIS* NUCLEOMODULINS

Several gut bacteria such as *Escherichia coli*, *Campylobacter jejuni,* and *Salmonella enterica* synthesize cytolethal distending toxins (Cdts), which are known to interfere with host cell cycle processes (66). Cdts comprise three subunits, CdtA, CdtB, and CdtC. CdtA and CdtC assist in tethering the toxin to the plasma membrane of target cells (23, 66). CdtB is a homolog of eukaryotic deoxyribonuclease I that translocates to the host nucleus, causing double-strand breaks in DNA, leading to cell death (66). CdtB from *E. coli, Aggregatibacter actinomycetemcomitans,* and *Haemophilus ducreyi* has been thoroughly researched to comprehend the mechanism of its translocation to the nucleus (23). In spite of a high degree of conservation in the homologs of the CdtB subunit, the location of the NLS might differ among these proteins. Two putative NLSs have been reported in *E. coli* CdtB (23, 66). Although mutations in either of these NLSs did not affect the DNase activity, they impaired the protein’s ability to access the host nucleus and induce cell cycle arrest (66). *C. jejuni* CdtB employs unknown mechanisms distinct from typical CdtB, as observed in other organisms (67).

Cycle inhibiting factor (Cif), also a well-studied cyclomodulin in *E. coli*, is a protease comprising cysteine, histidine, and a glutamine catalytic triad (68). It is injected into the host cell via T3SS and is primarily known to irreversibly block cell cycle progression. Inside the host nucleus, Cif deamidates NEDD8 (neuronal precursor cell-expressed developmentally downregulated protein 8) and induces accumulation of the cyclin-dependent kinase inhibitors p21 and p27 (68). NEDD8 is a ubiquitin-like protein that activates cullin-RING E3 ubiquitin ligase (CRL) by NEDDylating the cullin subunits (68, 69). This marks the proteins for ubiquitin-based degradation by the 26S proteasome. However, in case of *E. coli* infection, Cif interacts with NEDD8 and obstructs the cascade, leading to ubiquitin-mediated p21 and p27 degradation, thus halting the cell cycle at the G1/S and G2/M phases (34, 35, 68). Cif lacks a cNLS signal, and the mechanism of its transport to the host nucleus has not yet been characterized.

The third *E. coli* effector protein, EspF, lacks a classical nuclear/nucleolar localization signal but contains a 51-amino-acid domain located at the N-terminus, which aids in targeting it to the nucleolus. Intriguingly, EspF is first accumulated in the mitochondria, which then controls the scope of EspF’s nucleolar entry. However, the regulatory facet of EspF during the course of infection has been minimally investigated and requires further examination. Once in the nucleolus, EspF inflicts damage on the most abundant protein in the vicinity, nucleolin, thereby affecting ribosome biogenesis (36).

Tus and CjeCas9 are among the other nuclear-targeted proteins from *E. coli* and *C. jejuni,* respectively (31, 70). Tus proteins are terminator proteins that halt replication by binding to terminator sequences (71). *E. coli* Tus comprises an NLS-like sequence, ^227^KLKIKRPVK^235^, located at the C-terminus (70). The dual functionality of the Tus protein in modulating host cell signals and facilitating infection remains to be investigated in depth. The functional role of another nucleomodulin, colibactin from *E. coli*, in colorectal cancer has been well elucidated (72). Although colibactin is known to enter the nucleus and induce interstrand cross-links and double-strand breaks in DNA (73), the mechanism it employs to enter the nucleus remains unknown. *C. jejuni* protein CjeCas9 nuclease enters host cells via outer membrane vesicles and subsequently reaches the nucleus with the help of a BP cNLS, ^44^RRLARSARKRLARRKAR^60^, where it induces cell damage by degrading chromatin (31, 32).

Another nucleomodulin known to interfere with cell cycle processes is protease HRgpA (arginine-specific gingipain) from *Porphyromonas gingivalis* (50). Although *P. gingivalis* is an oral anaerobe that contributes to the pathogenesis of periodontitis, it is frequently observed to be translocated to the gut in patients with pancreatic cancer and rheumatoid arthritis (74–76). HRgpA has been reported to effectively translocate to the host cell nucleus *in vitro*, but its mechanism of translocation remains unclear (50).

## CHROMATIN ASSEMBLY INTERFERENCE BY *LISTERIA MONOCYTOGENES*

*Listeria monocytogenes* is an opportunistic pathogen that colonizes the gastrointestinal tract during listeriosis, an infectious foodborne disease (77). *L. monocytogenes* produces several virulence factors including NMs such as LntA, OrfX, and InlP (42, 43, 78). The entry of LntA into the host nucleus is speculated to be facilitated by a putative BP cNLS, determined *in silico*. LntA targets bromo adjacent homology domain containing protein 1 (BAHD1), a transcriptional repressor implicated in the formation of heterochromatin (42, 79). It interferes with chromatin assembly by preventing recruitment of BAHD1 to interferon-stimulated genes (ISGs), thereby inducing their expression, resulting in colonization of *L. monocytogenes* by modulating IFN- γ-mediated immune response (42). Downregulation of BAHD1 has been shown to decrease the chance of survival in the case of lung cancer patients, suggesting its importance during the infection (42, 80). Another nucleomodulin from *L. monocytogenes* is OrfX, which lacks cNLS, and the mechanisms employed for facilitating its nuclear entry are yet to be characterized (43). This nucleomodulin targets host nuclear zinc finger protein RybP (RING1- and YY1-binding protein), which is one of the critical proteins involved in developmental as well as proapoptotic pathways (43, 81). RybP is also known to be oncogenic in nature as it is involved in averting proteasome-mediated ubiquitination of tumor suppressor protein p53 by interacting with E3 ubiquitin-protein ligase MDM2 (43, 82). OrfX is positively regulated by PrfA, which is overexpressed during *L. monocytogenes* infection and is reported to activate other vital virulence proteins. Overall, the interaction of OrfX and RybP leads to dysfunctionality of macrophages in initiating oxidative burst, thus aiding in the intracellular survival of the bacteria (43). Additionally, the third nucleomodulin, Internalin P (InlP) from *L. monocytogenes,* targets nuclear speckles, which are dynamic structures constituting pre-mRNA splicing components (83). The specific mechanism by which InlP translocates to the nucleus remains unclear; however, once in the host nucleus, it specifically targets RBM5 (RNA-binding motif protein 5), an mRNA splicing regulator involved in cell cycle regulation and apoptosis (78, 83, 84).

## INDUCTION OF DNA METHYLATION BY *ACINETOBACTER BAUMANNII*

*Acinetobacter baumannii* is an opportunistic pathogen primarily involved in nosocomial infections where it colonizes the gut and often develops antimicrobial resistance (85). While 15 proteins from *A. baumannii* have been experimentally validated to target the host nucleus, the mechanism of action has been clearly elucidated only for two of these proteins, transposase (Tnp) and outer membrane protein A (AbOmpA) (29, 30). *A. baumannii* entry to the cytoplasm of host cells is facilitated by outer membrane vesicles. Once in the cytoplasm, Tnp is delivered to the host nucleus via MP cNLS ^225^RKRKRK^230^, which then contributes to epigenetic modifications in the host cell, leading to the downregulation of E-cadherin expression (29, 30, 85, 86). Tnp suppresses E-cadherin expression by inducing DNA methylation in the CpG islands of the promoter region of the gene (30). E-cadherin is a tumor suppressor gene, the loss of which, along with that of some other proteins, has been implicated in the initiation of epithelial mesenchymal transition (EMT) in cancer, a process that drives tumor metastasis (87–89). Another *A. baumannii* protein, OmpA, which is involved in apoptosis of epithelial cells, also targets the nuclear compartment of the host cell using an MP cNLS ^320^KTKEGRAMNRR^330^. Choi et al. (29) through site-directed mutagenesis studies demonstrated the significance of two lysine residues in the NLS that enable this nucleomodulin to enter the host nucleus (29, 86). Interestingly, OmpA has also been reported to translocate to the mitochondria, and this colocalization is suggested to be crucial for hindering the physiological state of the cell, eventually resulting in cell death (29).

## MASTER EPIGENETIC REGULATOR *MYCOBACTERIUM TUBERCULOSIS*

**Fig 2:**
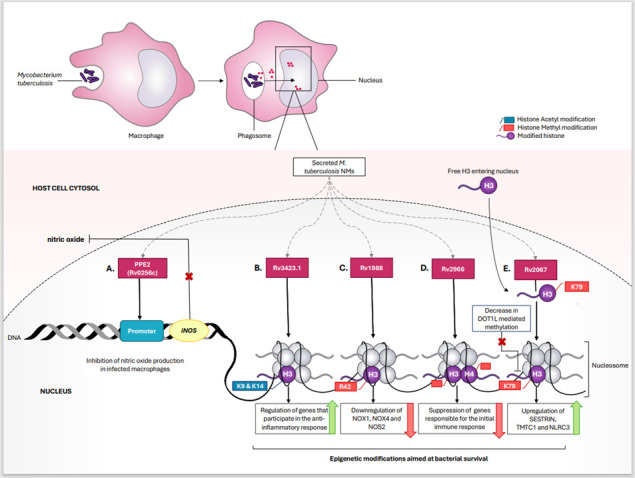
Nucleomodulins secreted by *M. tuberculosis* in phagosome escape into the cytosol followed by translocation to the nucleus. Once inside the macrophage nucleus, (A) Rv0256c binds to the iNOS promoter and inhibits iNOS transcription, resulting in reduced nitric oxide (NO) production. (B) Rv3423.1 acetylates H3 histone, leading to alteration of anti-inflammatory responses. (C) Rv1988 methylates the arginine residue at the 42 position of histone H3, thereby suppressing the expression of genes such as NOX1, NOX4, and nitric oxide synthase 2 (NOS2), which are involved in the production of reactive oxygen species (ROS). (D) Rv2966 methylates cytosine residues of histones H3 and H4 in a non-CpG context, resulting in downregulation of genes responsible for the initial immune response. (E) Rv2067 methylates nuclear-targeted non-nucleosomal H3 histone in cytosol as well as in the nucleus, thus downregulating the epigenetic activity of human methyltransferase DOT1L. This results in upregulation of SESTRIN, TMTC1, and NLRC3 and subsequent inhibition of caspase-8-dependent apoptosis.

*Mycobacterium tuberculosis* is a pathogen known to exhibit a range of evading mechanisms by hijacking host immune responses. Although a respiratory pathogen, it can translocate to the gut, particularly in the ileocecal region, leading to gastrointestinal tuberculosis (90). Rv1988, Rv2067c, Rv3423.1, Rv2966c, and Rv0256c are *M. tuberculosis* nucleomodulins that directly interfere with immune defense mechanisms by manipulating the host’s epigenetic landscape (44, 47–49). These mechanisms have been schematically depicted in Fig. 2. Rv1988 is a methyltransferase secreted via the Tat-secretion pathway in virulent mycobacterial strains. It dimethylates arginine in histone H3 (H3R42), resulting in suppression of genes encoding NADPH oxidases (NOX1 and NOX4) and nitric oxide synthase (NOS2) (44). This allows mycobacteria to overcome the initial line of defense, replicate effectively within cells, and spread the infection (44, 91). Yaseen et al. (44) demonstrated that the nuclear targeting of Rv1988 is facilitated by an arginine-rich region spanning amino acids from 137 to 179 (^137^RR^139^, ^152^RR^154^, and ^169^RRRK^173^) located at the C-terminus (44). Further, the study highlights the propensity of Rv1988 toward methylation of non-tail core histone H3-arginine H3R42, which is usually not a target for methylation by mammalian histone methyltransferases (44).

Rv2067c is another methyltransferase, which enters the nucleus with the help of 30 amino acid residues located in the C-terminus (48). It is capable of methylating free, non-nucleosomal histone H3 at the K79 position (H3K79) in both cytoplasmic as well as nuclear environments of the macrophages. Interestingly, Rv2067c suppresses the expression of the host methyltransferase DOT1L (Disruptor of Telomeric silencing 1-like), which is also directed toward the same methylation site as Rv2067c but in a nucleosomal H3 (48). The proclivity of both methyltransferases to target the same site in H3 varies depending on the nucleosomal context due to significant structural differences between the two methyltransferases. Rv2067 is a homodimer that contains N-terminal SAM-binding catalytic domain (CD), a dimerization domain (DD), and a C-terminal domain (CTD), whereas DOT1L is a monomer with distinctly different domains from Rv2067c, except for the shared Class I MTase fold (48). Furthermore, the binding of DOT1L induces a conformational change in the H3K79 loop, moving H3K79 from an inaccessible position into the active site of DOT1L, thereby aiding the process of methylation. This epigenetic marker upregulates the expression of genes encoding proteins such as TMTC1, SESTRIN3, and NLRC3, collectively shielding pathogens from host immune responses (48).

A third methyltransferase, Rv2966c, was found in the nucleus of THP1 cells *in vivo* and was further determined to bind and methylate non-CpG (distinctly CpA and CpT) dinucleotides (47). A stretch of 20 amino acids located in the C-terminal region, comprising three arginine (at positions 171, 177, and 183) and one threonine residue, was suggested to be the signal sequence of Rv2966c. In addition to arginine, the mutation in T176 also affected the nuclear targeting of the protein. Based on the interactions between nucleophosmin (NPM1) and Rv2966 through *in vitro* experiments, Sharma et al. (47) suggested the potential role of the NPM1 protein in the nuclear targeting of Rv2966. Post-translational modifications, such as phosphorylation, by PknB were reported to increase the DNA binding and methylating activity of Rv2966c. Rv2966c was also found to bind to specific gene regions including one within H2AFY2, a gene that codes for macro histone and G protein-coupled receptor kinase 5 (GRK5). Further, in support of “Rv2966-GRK5”-mediated pathogenesis, GRK5 has been reported to be significantly downregulated in *Mycobacterium*-infected THP1 macrophages and has previously been shown to interact with DNA and proteins other than GPCR in the nucleus (47).

Rv3423.1 is another mycobacterial epigenetic regulator of gene expression during infection. Rv3423.1 is a histone acetyltransferase and is reported to acetylate histone H3 at the K9/K14 positions. Given the small size of this protein (8.4 kDa) and lack of functional NLS, it is speculated that it may diffuse through nuclear pores or hijack eukaryotic nuclear-targeted protein to gain access to the host nucleus (49). The role of this protein once it is in the nucleus is not yet well-elucidated. However, given the nature of this enzyme, it is suggested that it may be involved in escaping initial host immune responses, thereby prolonging the bacterial intracellular survival period inside macrophages (49, 92).

Furthermore, another nucleomodulin secreted by *M. tuberculosis* is Rv0256c (PPE2), which comprises proline-glutamate (PE) and proline-proline-glutamate (PPE) residues. The presence of functional MP NLS located at the C-terminal facilitates nuclear entry of PPE2 mediated via the classical importin α/β pathway, mimicking eukaryotic protein nuclear transport. Once within the nucleus, PPE2 inhibits nitric oxide production in infected macrophages as it binds to the iNOS promoter and leads to masking the GATA-1 binding sites. This inhibition renders the macrophages dysfunctional, thereby conferring an intracellular survival advantage to mycobacteria (45, 46).

## DISCUSSION

The human gut is home to microbial communities important for maintenance of a balanced environment. An imbalance in these communities often leads to a conducive environment for the colonization of pathogens and the development of disease conditions. Studies have shown that this dysbiosis in the microbiome is associated with modulation in host gene expression, cell signaling pathways, chromatin dynamics, and even epigenetic regulation. Bacteria can utilize multiple ways to affect the host homeostasis, including utilizing their metabolites and membrane proteins to evade host immune response and influence regulatory and signaling pathways. In addition, bacteria utilize various effector proteins to reprogram host cells using different mechanisms of action. One such class of proteins includes nucleomodulins (NMs), which attack the most crucial eukaryotic cell organelle, the nucleus. Bacterial NMs have evolved to possess classical signal sequences that facilitate nuclear localization in the host cells, thereby mimicking eukaryotic nuclear proteins. Translocation of these NMs into the host cell nucleus has been experimentally validated to induce a multitude of cell cycle alterations, including epigenetic rewiring, that aids in establishing a compatible niche for the pathogen to replicate and spread. As the functioning of NMs involves an interaction with an array of host proteins in the gut environment, NMs play a crucial role in shaping the dynamics of the host-microbiome interaction. The current review presents a comprehensive collation of NM-dependent strategies, primarily employed by opportunistic gut pathogens, which would help in designing future experiments toward gaining better insights into the host-pathogen (as well as host-microbiome) interaction. In addition to classical NLS-dependent translocation, the present review discusses various other nuclear translocation mechanisms reported in recent studies.

Apart from the classical and non-classical NMs discussed in the current review, *Enterococcus faecalis* presents a distinct case wherein the whole bacterial cells are congregated in the perinuclear region surrounding the nucleus (33). *E. faecalis* is a common opportunistic pathogen, which can cause antibiotic-resistant infections and has also been frequently linked to colorectal cancer (93, 94). *E. faecalis* was found to replicate intracellularly in both immune and non-immune cells *in vitro*. The initial entry of the pathogen into the host cells is facilitated by macropinocytosis, followed by the downregulation of the GTPase Rab7. This process enables the bacteria to negatively regulate endosome-lysosome fusion and replicate within the cells, thereby prolonging the infection period (33).

Experimental identification of nucleomodulins is usually performed by tracking their nuclear localization using fluorescence-based techniques, the resolution of which depends on the expression levels. Such identification can be challenging during certain stages of infection as NM expression is dominated by the expression of primary virulent factors. For instance, as previously discussed in the case of *H. pylori*, the nucleomodulin Omp18 regulates the expression of primary virulence factors CagA and NapA, thus escaping early host inflammatory responses aimed at eliminating the pathogen (59, 60). Therefore, it is essential to monitor the expression levels of both nucleomodulins and primary virulence factors over the course of infection, toward evaluating the complex network of these proteins driving the pathogenesis. The comprehensive collection of nucleomodulins with signal sequence information, provided in this review, can help in building robust methods capable of predicting diverse categories of bacterial NMs.

Given the ability of bacterial nucleomodulins to interact with host cell DNA and alter nuclear functions, including gene expression, recent literature suggests their diverse roles in exacerbation of various infectious diseases and cancer. As mentioned earlier, NMs from *H. pylori* and *E. coli* have been reported to be upregulated in cancer patients (61, 62, 72, 95). Further, one of the NMs from *E. coli,* colibactin, was found to induce specific mutation at the DNA damage site in infected human colorectal cancer cells (73, 95). Overall, involvement of bacterial infection in cancer pathophysiology is being increasingly acknowledged. Nucleomodulins, being a suspected key driver of the “bacterial infection-cancer axis,” demands focused studies toward their potential utilization in diagnostic and therapeutic regimes. Therefore, accounting for both realms of host physiology and interacting microbiome is important for improving cancer therapy as well as prognosis.

## AFTER EPUB

*[This article was published on 18 July 2025 with typographical errors in the Introduction and Discussion. The errors were corrected in the current version, posted on 23 July 2025.]*
